# A bi-kinase module sensitizes and potentiates plant immune signaling

**DOI:** 10.1126/sciadv.adt9804

**Published:** 2025-01-24

**Authors:** Philipp Köster, Gefeng He, Changyun Liu, Qiuyan Dong, Katarina Hake, Ina Schmitz-Thom, Paulina Heinkow, Jürgen Eirich, Lukas Wallrad, Kenji Hashimoto, Stefanie Schültke, Iris Finkemeier, Tina Romeis, Jörg Kudla

**Affiliations:** ^1^ Institut für Biologie und Biotechnologie der Pflanzen, Universität Münster, Münster, Germany.; ^2^ Dahlem Centre of Plant Sciences, Institut für Biologie, Freie Universität Berlin, Berlin, Germany.; ^3^ Leibniz-Institut für Pflanzenbiochemie, Halle, Germany.

## Abstract

Systemic signaling is an essential hallmark of multicellular life. Pathogen encounter occurs locally but triggers organ-scale and organismic immune responses. In plants, elicitor perception provokes systemically expanding Ca^2+^ and H_2_O_2_ signals conferring immunity. Here, we identify a Ca^2+^ sensing bi-kinase module as becoming super-activated through mutual phosphorylation and as imposing synergistically enhanced NADPH oxidase activation. A combined two-layer bi-kinase/substrate phospho-code allows for sensitized signaling initiation already by near-resting elevations of Ca^2+^ concentration. Subsequently, it facilitates further signal wave proliferation with minimal Ca^2+^ amplitude requirement, triggering protective defense responses throughout the plant. Our study reveals how plants build and perpetuate trans-cellular immune signal proliferation while avoiding disturbance of ongoing cellular signaling along the path of response dissemination.

## Teaser

Mutual activation of a Ca^2+^ sensing bi-kinase module potentiates NADPH oxidase activation to facilitate systemic immunity.

## INTRODUCTION

Initial to any immune response is pathogen perception by specifically committed pattern recognition receptors (PRRs) (*1*). Elicitor sensing in the primary infected cells prompts auto- and transphosphorylation of involved receptor kinase (RK) and receptor-like protein (RLP) complexes that subsequently trigger local and systemically spreading Ca^2+^ and reactive oxygen species (ROS) signals, of which the latter can be produced through activation of NADPH (reduced form of nicotinamide adenine dinucleotide phosphate) oxidases (NOXs) (*2*, *3*). NOX-dependent physiological generation of ROS for manifestation of systemic innate immunity is highly conserved across virtually all multicellular life (*4*). However, how distal cell-to-cell/trans-cellular propagation of these second messenger signals is perpetuated in the absence of elicitor stimulation remains largely enigmatic (*5*–*7*).

In plants, perception of molecular patterns by plasma membrane (PM)–localized PRRs induces responses termed pattern triggered immunity (PTI). In *Arabidopsis thaliana* (hereafter Arabidopsis), perception of the bacterial elicitor peptide flg22 through the RK FLS2 and its coreceptor BAK1 leads to phosphorylation and activation of the receptor-like cytoplasmic kinase (RLCK) BIK1 (*8*, *9*). BIK1 functions in initiating subsequent Ca^2+^ and ROS signals (*10*, *11*). BIK1 and the Ca^2+^-activated kinase CPK5 directly phosphorylate the NOX RBOHD, thereby triggering apoplastic formation of superoxide (O_2_^−^) and consequently H_2_O_2_ (*12*–*14*). Accordingly, *rbohd*, *bik1*, and *cpk5* mutants exhibit compromised local and, as consequence, impaired systemic immune responses (*12*–*15*). Ca^2+^ signals are directly decoded through Ca^2+^-dependent protein kinases (CPKs) and through a network of calcineurin B–like (CBL) Ca^2+^ sensor proteins and CBL-interacting protein kinases (CIPKs) into downstream responses. Moreover, calmodulins (CAMs) and CAM-like proteins contribute to the deciphering of Ca^2+^ signals in plants (*16*). CBL/CIPK complexes regulate a multitude of crucial transcription factors, ion channels, and transporters and also activate the NOXs RBOHC and RBOHF (*17*–*19*). However, if and how CBL/CIPK Ca^2+^ signal sensors/decoders also function in systemic immunity signaling remain to be addressed.

## RESULTS

### A local Ca^2+^/phosphorylation switch triggers systemic innate immunity

**Fig. 1.**
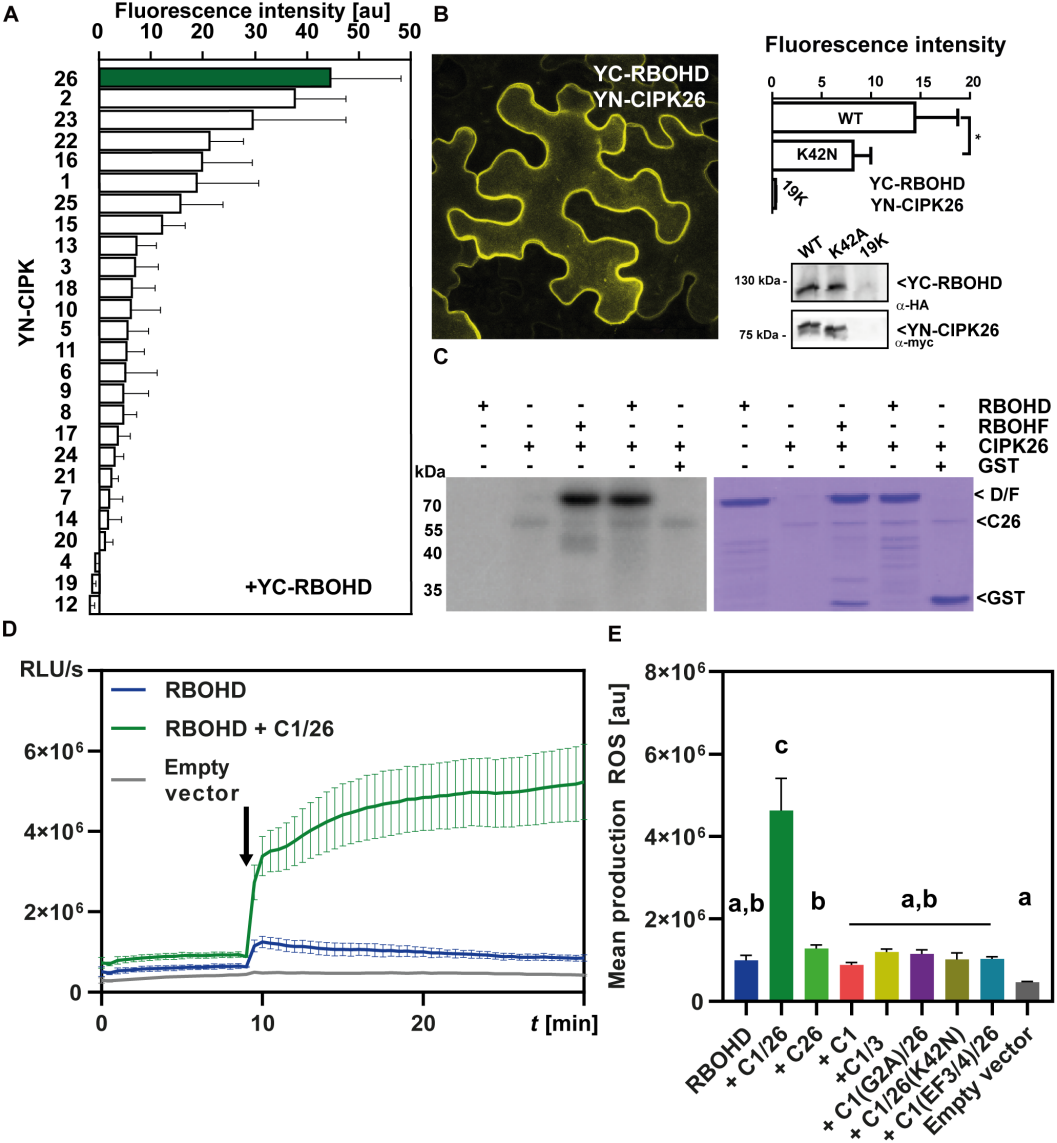
CBL1/CIPK26 associates with and activates RBOHD. (**A**) BiFC-based interaction screen combining each of the 26 CIPKs from Arabidopsis with RBOHD. Bars indicate mean fluorescence intensity of six images; error bars indicate SE. (**B**) BiFC analysis in *N. benthamiana* leaf epidermis cells. The image depicts a 20-stack maximum projection. Kinase activity–deficient CIPK26^K42N^ exhibits reduced interaction with RBOHD. Leaves infiltrated solely with the 19K helper strain were used as background control. The bar plot illustrates mean fluorescence intensity of six images; error bars indicate SE. Western analysis with antibodies specific for c-myc and hemagglutinin (HA) tags confirmed faithful expression of YC-RBOHD, YN-CIPK26, and YN-CIPK26^K42N^. (**C**) In vitro kinase assays reveal phosphorylation of the RBOHD N terminus by CIPK26. The RBOHF N terminus served as positive control. GST protein was not phosphorylated by CIPK26. Shown are autoradiogram and CBB-stained gel. (**D**) CBL1/CIPK26 complexes activate RBOHD-dependent ROS production in HEK293T cells. Ca^2+^ influx into cells was initiated after 10 min (indicated by an arrow). ROS production was quantified as relative light units (RLU). Error bars indicate SD. Each data point represents the mean of three wells analyzed in parallel. (**E**) RBOHD activation requires CBL1/CIPK26 complex formation, CIPK26 kinase activity, as well as CBL1 plasma membrane targeting and Ca^2+^ binding (see ROS curves in fig. S1); one-way analysis of variance (ANOVA); Tukey’s posttest; letters denote statistical differences between samples. Error bars indicate SD. Each data point represents the mean of three wells analyzed in parallel. D, RBOHD; C1/26, CBL1/CIPK26 complex; C1, CBL1; C26(K42N), CIPK26^K42N^; C1/3, CBL1/CIPK3 complex; C1(G2A), CBL1^G2A^; C1(EF3/4), CBL1 EF3/4.

We pursued a bimolecular fluorescence complementation (BiFC)–based interaction screen, combining all 26 CIPKs from Arabidopsis with RBOHD, and identified CIPK26 as most strongly interacting with RBOHD (Fig. 1A). Kinase-RBOHD interaction occurred at the PM, and active CIPK26 displayed stronger interaction than a kinase-inactive variant (Fig. 1B). Recombinant CIPK26 phosphorylated the N-terminal domain of RBOHD with similar efficiency as the known CIPK26 substrate RBOHF (Fig. 1C) (*17*). To address if and how CIPK26 regulates RBOHD activity in a cellular context, we used reconstitution of the plant Ca^2+^ signaling/ROS generation module in human embryonic kidney (HEK) 293T cells (Fig. 1D). While expression of RBOHD alone only allowed for minor ROS production, coexpression of CBL1/CIPK26 with RBOHD conferred readily detectable ROS production already at basal Ca^2+^ concentration, which was fivefold enhanced upon elicitation of Ca^2+^ signals. Moreover, CIPK2 and CIPK23, which also displayed considerable interaction in BiFC assays, but not CIPK22 and CIPK16, which displayed roughly half BiFC signal intensity with RBOHD, could activate RBOHD in HEK293T cells. Also, the most closely related kinase CIPK3, which only weakly interacted in BiFC assays, did not evoke RBOHD activation (fig. S1). RBOHD activation strictly depended on kinase activity and the presence of both the CBL1 Ca^2+^ sensor and the kinase. Moreover, impairing Ca^2+^ binding of CBL1 by mutating critical EF-hands or abrogating CBL1/CIPK26 PM targeting by mutating the CBL1 myristoylation motif attenuated ROS production back to levels observed by expression of RBOHD alone. Also, the Ca^2+^ sensor CBL9, which is closely related to CBL1, could in combination with CIPK26 provoke RBOHD activation, although to a much lesser extent than CBL1 (Fig. 1E and fig. S1). Together, these data identify CBL1/CIPK26 as a Ca^2+^ sensor/kinase module that can bring about Ca^2+^-dependent ROS generation through RBOHD activation.

### CBL1/CIPK26/CPK5 collectively confer synergistic RBOHD activation and systemic PTI

**Fig. 2.**
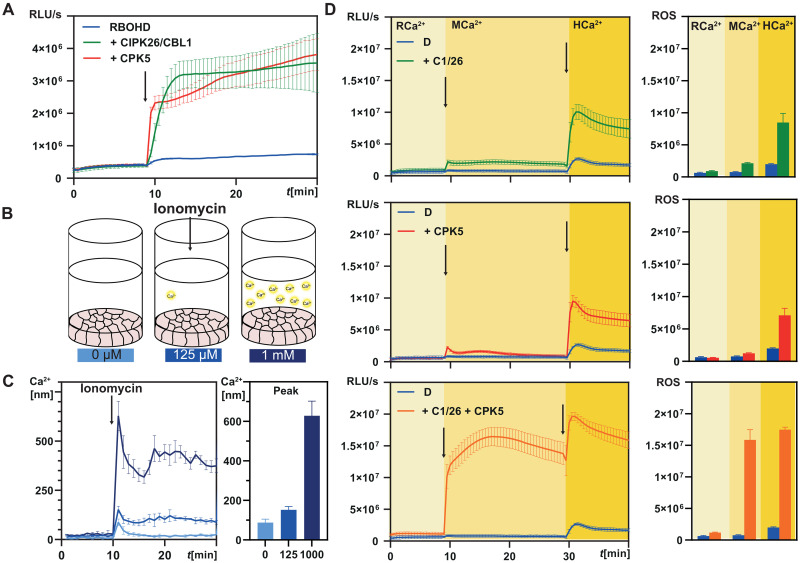
Combined activity of CPK5 and CBL1/CIPK26 triggers sensitized synergistic activation of RBOHD. (**A**) CPK5 and CBL1/CIPK26 similarly activate RBOHD in HEK293T cells. ROS production was quantified as relative light units (RLU). Error bars indicate SD. Each data point represents the mean of three wells analyzed in parallel. (**B**) Graphical summary of the experimental setup for controlled quantitative modulation of cytoplasmic Ca^2+^ in HEK293T cells. (**C**) Fura-2–based Ca^2+^ concentration determination reveals MCa^2+^ elevation to 150 nM in response to 0.125 mM external Ca^2+^ and HCa^2+^ elevation to 600 nM. The numbers below the bars indicate the external Ca^2+^ concentration in the medium as also indicated in (B). The color code of the graphs refers to these external Ca^2+^ concentrations. Arrow indicates injection of 1 μM ionomycin. Bar plots indicate peak Ca^2+^ elevation. Error bars indicate SD. Each data point represents the mean of three wells analyzed in parallel. (**D**) Coexpression of RBOHD with CBL1/CIPK26 and CPK5 triggers sensitized and synergistic activation of ROS production already at MCa^2+^, which is further elevated at HCa^2+^. ROS production was quantified as RLU. Error bars indicate SD. Each data point represents the mean of six wells analyzed in parallel. Bar plots display mean ROS production in 5-min time intervals (RCa^2+^: minute 2.5 to 7.5; MCa^2+^: minute 17.5 to 22.5; HCa^2+^: minute 32.5 to 37.5). Error bars indicate SEM.

Currently, the reasons for the coexistence of the two distinct Ca^2+^-decoding kinase networks, the CBL/CIPK system and the CPKs, remain enigmatic (*16*). The identification of CPK5 and CIPK26 as kinases phosphorylating RBOHD enabled us to investigate the functional interrelation of these two kinase classes. Coexpression of CPK5 with RBOHD in HEK293T cells revealed a similar degree of Ca^2+^-dependent RBOHD activation as conferred by CBL1/CIPK26 (Fig. 2A).

We next sought to assess the individual roles of both kinases in RBOHD activation and to quantitatively dissect their Ca^2+^ dependence. To this end, we devised a synthetic cellular Ca^2+^ signaling reconstitution system that allowed for quantitatively controlling the amplitude of Ca^2+^ signals and thereby for faithful parameter control of regulatory circuits in HEK293T cells (Fig. 2B). While ionomycin application at external Ca^2+^ concentrations of 0.125 mM triggered moderate cellular Ca^2+^ signals of ~150 nM (±15) (moderate Ca^2+^, MCa^2+^), external Ca^2+^ concentrations of 1 mM elicited intracellular Ca^2+^ signals with an amplitude of 630 nM (±60) (high Ca^2+^, HCa^2+^) (Fig. 2C). Application of this protocol to cells either expressing RBOHD alone or RBOHD combined with CBL1/CIPK26 or CPK5 uncovered a clear correlation between cellular Ca^2+^ concentration and resulting H_2_O_2_ production indicative for RBOHD activity (Fig. 2). For both CPK5 and CBL1/CIPK26, when expressed individually with RBOHD, we observed that stepwise increases in Ca^2+^ concentration incrementally increased RBOHD activity to comparable extent (Fig. 2D). While MCa^2+^ triggered a two to three times increase in H_2_O_2_ generation either with CBL1-CIPK26 or with CPK5, HCa^2+^ caused a drastically enhanced H_2_O_2_ production more than 15-fold higher compared to resting Ca^2+^ concentration (RCa^2+^, 87 nM). We next elucidated the combined effect of CBL/CIPK- and CPK-mediated regulation on RBOHD activity by simultaneously coexpressing RBOHD with CBL1/CIPK26 and CPK5 (Fig. 2D and fig. S2). HCa^2+^ triggered a notably enhanced ROS production that was doubled compared to activation by either CBL1/CIPK26 or CPK5 alone. Most remarkably, already at MCa^2+^, the combined function of both kinases caused eightfold increased RBOHD activity indicated by ROS production to a level that the individual kinases could not evoke even under HCa^2+^ conditions. This synergistic activation was not a consequence of increased quantity, since duplication of the amount of individually transfected kinases did not evoke such a marked activation of RBOHD (fig. S2). Collectively, these findings uncover a marked synergistic effect of combined CPK and CBL/CIPK function on the activity of RBOHD. Moreover, these results imply simultaneous sensitization to Ca^2+^ concentration as well as potentiation of phosphorylation-mediated RBOHD activation. Potential mechanisms could involve synergistic interdependences between the impact of Ca^2+^ binding to the EF-hands of CBL1, CPK5, and RBOHD (providing a combinatory Ca^2+^ code) and, on the other hand, enhanced phosphorylation efficiency of p-sites in RBOHD and/or in CBL1/CIPK26/CPK5 (providing a complementary phospho-code).

**Fig. 3.**
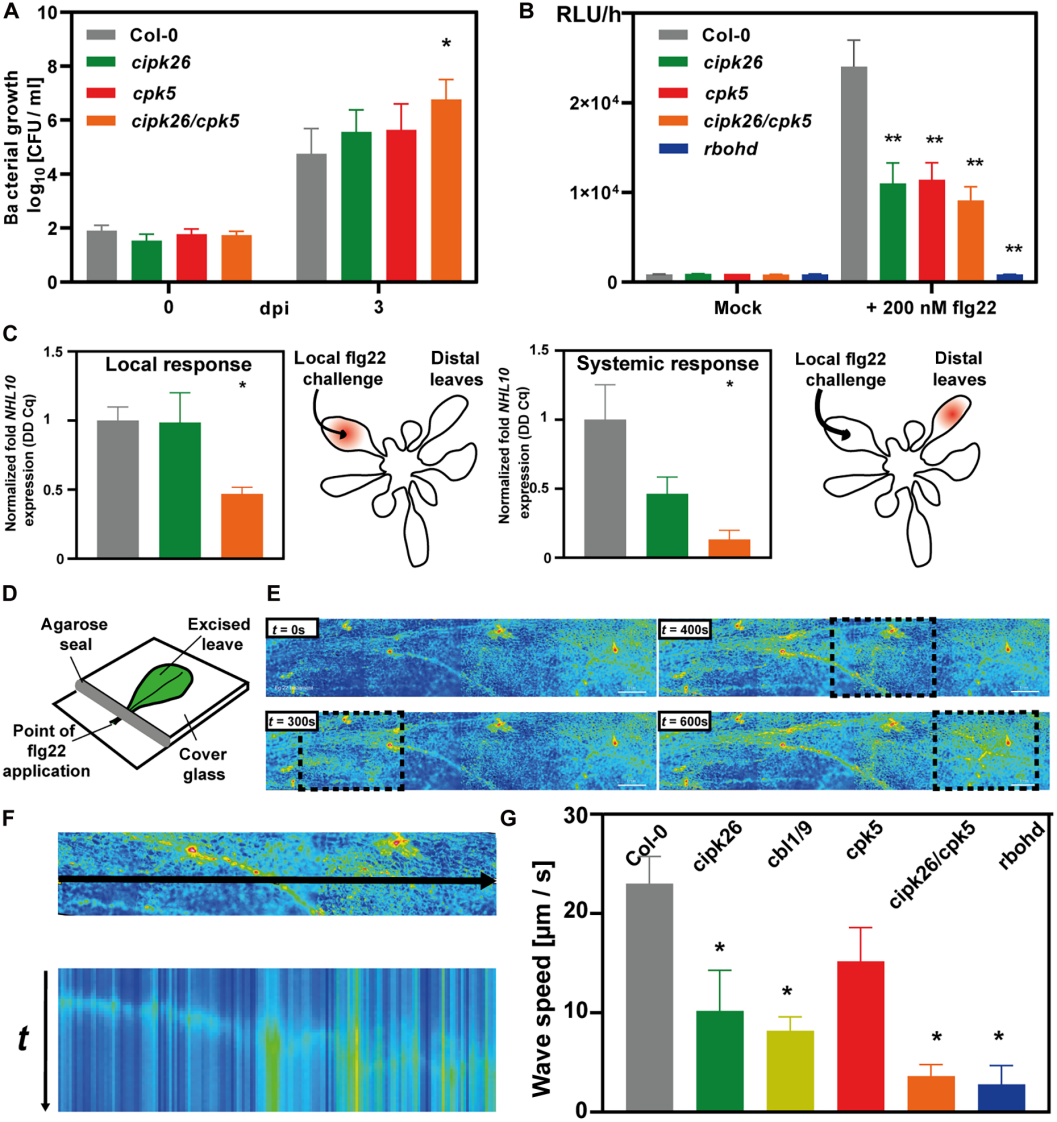
The CBL/CIPK26/CPK5/RBOHD module confers initiation and propagation of immune signaling. (**A**) Loss of CIPK26 and/or CPK5 function renders Arabidopsis plants susceptible to *P. syringae*. Bacterial growth in the indicated genotypes after inoculation with *Pst* DC3000. dpi, day post-inoculation; CFU, colony-forming units; error bars, SEM (*n* ≥ 9); one-way ANOVA; Tukey posttest; asterisks denote statistical differences to Col-0; *P < 0.05. (**B**) CIPK26 and CPK5 are required for flg22-induced ROS burst. ROS production was determined via a luminol-based assay. Error bars, SEM (*n* ≥ 8); one-way ANOVA; Dunnett’s posttest; asterisks denote statistical differences to Col-0; **P < 0.01. (**C**) Flg22-induced *NHL10* expression induction requires CIPK26 function. Forty-five minutes after flg22 injection, *NHL10* expression was quantified by qRT-PCR in the indicated leaf. The color code for genotypes is as in (A). Error bars, SEM (*n* ≥ 7); Student’s *t* test; *P < 0.05. (**D**) Depiction of the setup for organ-scale Ca^2+^ analysis in leaves. Leaves of plants expressing Ca^2+^ reporter proteins were detached; 24 hours later, flg22 was added to the petioles. Ca^2+^ signals in the leaf blade were monitored by fluorescence microscopy, and their speed was measured. (**E**) R-GECO fluorescence in Arabidopsis Col-0 at different time points after flg22 application. Boxes highlight the progressing Ca^2+^ wave. (**F**) Overview picture with the line-type ROI spanning the leaf blade (top) and kymogram (bottom) displaying the wave front propagation along the ROI. (**G**) Speed of Ca^2+^ waves in indicated genotypes. *n* > 5; error bars indicate SEM. One-way ANOVA; Tukey’s posttest; asterisks denote statistical differences to Col-0.

To address the physiological relevance of CBL1/CIPK26/CPK5-dependent RBOHD activation in immunity, we isolated the respective mutants and characterized their PTI responses and pathogen resistance. Scoring of *Pseudomonas syringae* DC3000 proliferation 3 days after infiltration indicated similarly increased bacteria abundance in *cipk26* and *cpk5* leaves and revealed substantially further enhanced bacterial proliferation in leaves of *cipk26/cpk5* (Fig. 3A). Compared to wild type (WT), flg22 application evoked a clearly reduced ROS accumulation in directly exposed leaf discs of *cpk5* as well as of two independent *cipk26* alleles (Fig. 3B and fig. S3). This local immune response was not further reduced in *cipk26/cpk5*, but fully abolished in *rbohD*. Flg22 application triggers induction of the marker gene *NHL10* locally, but also in distal leaves that have not experienced direct elicitor exposure (*14*). We found that local *NHL10* up-regulation in flg22-challenged leaves was not impaired in *cipk26* but substantially reduced in *cipk26/cpk5* (Fig. 3C). Moreover, these analyses confirmed the reported contribution of CPK5 to local NHL10 induction (*14*). In stark difference, loss of CIPK26 notably reduced *NHL10* up-regulation in distal leaves, and in *cipk26/cpk5*, this response was almost abolished. Collectively, these in planta investigations reveal an important individual role of CPK5 and CIPK26 in pathogen resistance and establish the indispensable requirement of their combined function for systemic immune signaling.

We next sought to use the flg22-triggered systemic Ca^2+^ wave as a proxy to illuminate the role of Ca^2+^/phosphorylation-mediated RBOH activation in systemic signaling. To this end, we devised a systemic bioimaging assay for whole detached leaves based on flg22 application onto petioles and Ca^2+^ reporter–based monitoring of the resulting Ca^2+^ waves throughout the leaf blade (Fig. 3D). In WT, application of flg22 induced a Ca^2+^ wave initiating from the leaf basis, subsequently expanding to the whole width of the leaf, and forming a front that traveled toward the leaf tip (Fig. 3, E and F, and movie S1). The amplitude (signal intensity) of this wave was reproducibly clearly less pronounced than that of recently described Ca^2+^ waves, e.g., in response to NaCl stress (*20*). We determined a speed of 21 ± 5 (μm/s) (averaged over the length of the leaf) for systemic Ca^2+^ signal propagation through epidermal cells in WT (Fig. 3F). The speed of this organ-scale flg22 response is substantially slower compared to 200 to 1000 μm/s that have been determined for systemic electrical, ROS, or Ca^2+^ signals propagating through vascular bundles in response to other stimuli (*20*–*28*). Therefore, this value likely defines the cell-to-cell transcellular propagation velocity of elicitor-triggered Ca^2+^/ROS signals. By increasing the resolution of our analysis and focusing comparatively on individual regions of interest (ROIs) in the basal as well as in a distal position of the leave, we noticed that the intensity and velocity of this systemic signal steadily decreased with distance (fig. S4). In *rbohd*, wave formation and propagation was hardly detectable, corroborating the essential role of this NOX in cell-to-cell communication (Fig. 3G). Accordingly, Ca^2+^ signal intensity was close to the detection limit in both basal and distal regions (fig. S4). In individual mutants of CPK5, CIPK26, or CBL1/9 (the two Ca^2+^ sensors that activate CIPK26), the speed of the mobile Ca^2+^ signal was substantially reduced (Fig. 3G). Notably, impairment of CIPK-mediated phosphorylation exerted a stronger effect than compromised CPK phosphorylation. Strikingly, in *cpk5*/*cipk26*, systemic Ca^2+^ signal propagation was similarly collapsed as in *rbohd*. Also, the signal amplitude of the propagating Ca^2+^ signal was differentially affected in the different mutants (fig. S4). While *cipk26* and *cpk5* displayed strongly reduced but clearly discernible Ca^2+^ signals in basal ROIs, their intensity was further dramatically diminished in *cipk26*/*cpk5.* Notably, the decline of signal speed and amplitude, when comparing these parameters in basal and distal regions of the leaves, was shared with all genotypes and correlated with their Ca^2+^ signal speed/intensity in the respective basal ROI. Collectively, these findings uncover an absolute requirement of Ca^2+^-dependent RBOHD phosphorylation for effective systemic Ca^2+^ signal formation and propagation. Moreover, since CPK and CBL/CIPK activity toward RBOHD are strictly Ca^2+^ dependent, we also conclude that this mobile Ca^2+^ signal is essential for forming the elicitor-triggered ROS wave and for evoking distal transcriptional responses like *NHL10* induction.

### Mutual CPK5-CIPK26 phosphorylation facilitates Ca^2+^-sensitized and potentiated ROS signaling

**Fig. 4.**
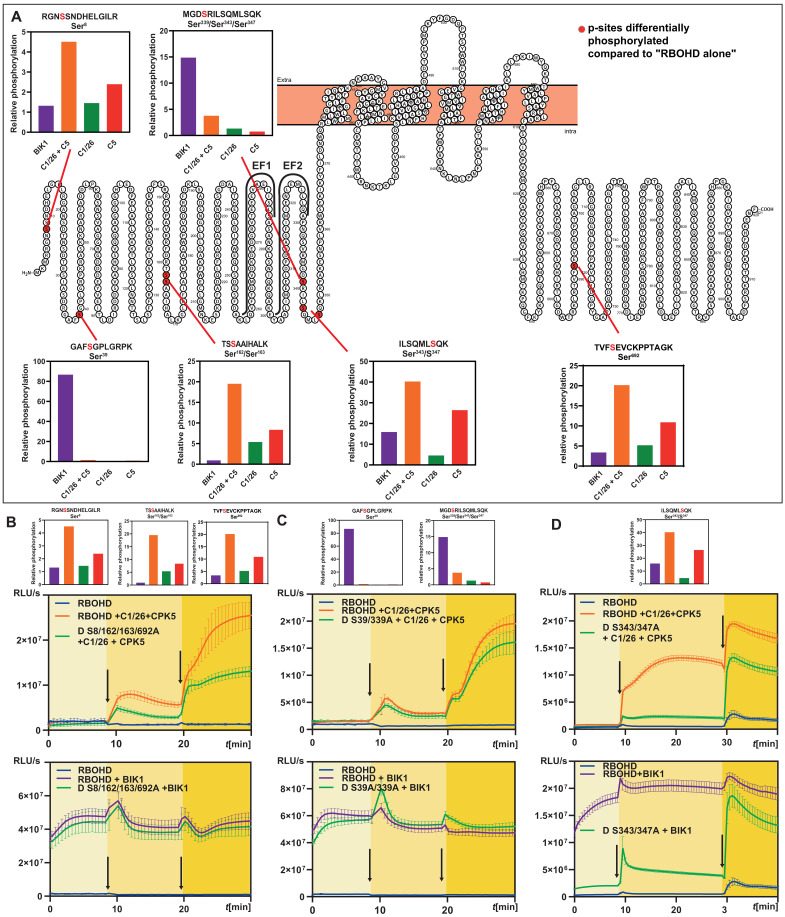
Ca^2+^-independent phosphorylation through BIK1 and Ca^2+^-dependent phosphorylation through CBL1/CIPK26 and CPK5 provide alternative routes for RBOHD activation. (**A**) Quantitative presentation and schematic depiction of p-sites identified as being differentially phosphorylated in response to moderate Ca^2+^ signals (MCa^2+^) in HEK293T cells expressing the indicated kinase combinations (compared to cells expressing RBOHD alone; bars indicate mean phosphorylation intensity of four replicates). (**B**) Mutation of the p-sites targeted by the Ca^2+^-dependent kinases (S8/162/163/692A) impairs activation of RBOHD by CPIK26/CPK5 but not by BIK1 in HEK293T cells. ROS production was quantified as relative light units (RLU). Error bars indicate SD. Each data point represents the mean of five wells analyzed in parallel. (**C**) Mutation of the p-sites targeted by BIK1 (S39/339A) does not impair activation of RBOHD in HEK293T cells. ROS production was quantified as RLU. Error bars indicate SD. Each data point represents the mean of five wells analyzed in parallel. (**D**) Mutation of the p-sites shared by CIPK26/CPK5 and BIK1 (S343/347A) renders BIK1-mediated RBOHD activation Ca^2+^ dependent and abolish Ca^2+^ phosphorylation–dependent activation of RBOHD. ROS production was quantified as RLU. Error bars indicate SD. Each data point represents the mean of six wells analyzed in parallel.

To dissect the mechanistic principles underlying the sensitized and potentiated activation of RBOHD, we combined control of cytoplasmic Ca^2+^ concentration with mass spectrometric phosphorylation site identification and quantitation. To cover the conditions of sensitized hyperactivation of RBOHD, HEK293T cells expressing RBOHD alone, RBOHD combined with individual CPK5 or CBL1/CIPK26 or BIK1, or RBOHD together with CBL1/CIPK26 and CPK5 were ionomycin-treated to evoke MCa^2+^ cytoplasmic Ca^2+^ signals (150 nM). Then, total protein was extracted and subjected to phospho-proteomic analysis. This approach identified seven serine and one threonine in RBOHD located on six distinct peptides (hereafter “p-sites”) as being principally phosphorylated upon kinase exposure (Fig. 4A and table S1). One p-site was almost exclusively targeted by BIK1 (Ser^39^), while the other p-sites were targeted by all three kinases to varying degree. Quantitative evaluation of the phosphorylation profile defined three distinct target site groups: (i) preferentially BIK1-addressed p-sites (Ca^2+^-independent: Ser^39^; Ser^339^), (ii) preferentially CBL1/CIPK26/CPK5-addressed p-sites (Ca^2+^-dependent: Ser^8^; Ser^162/163^; Ser^692^), and (iii) p-sites concurrently targeted by BIK1 as well as CBL1/CIPK26/CPK5 (Ser^343/347^). To address the functional relevance of Ca^2+^-dependent phosphorylation, we elucidated the activatability of RBOHD^Ser8/162/163/692A^ by CBL1/CIPK26/CPK5. Notably, in both MCa^2+^ and HCa^2+^ conditions, H_2_O_2_ production conferred by RBOHD^Ser8/162/163/692A^ was dramatically reduced to less than half of that of RBOHD (Fig. 4B). In contrast, this mutant version of RBOHD was not impaired in BIK1-mediated activation, thereby establishing the independent relevance of Ca^2+^-dependent phosphorylation for RBOHD activity modulation (Fig. 4B). Somewhat unexpectedly, mutation of BIK1-specific residues (RBOHD^S39/339A^) did not affect RBOHD activatability by BIK1 or CBL1/CIPK26/CPK5, revealing that modification (or modification-preventing mutation) of these p-sites is dispensable for RBOHD activity modulation (Fig. 4C). We discovered a surprising complex ROS generation pattern when analyzing the impact of the shared p-sites (RBOHD^S343/347A^). When RBOHD^S343/347A^ was combined with CBL1/CIPK26/CPK5, ROS production at HCa^2+^ (630 nM) was reduced by about 40% to a level produced by nonmutated RBOHD at MCa^2+^ (150 nM). Even more strikingly, at MCa^2+^, RBOHD^S343/347A^ produced only as much H_2_O_2_ as RBOHD alone (without any kinase) would produce at HCa^2+^ (Fig. 4D). This identifies Ser^343/347^ as mechanistic switch essential for allowing sensitized activation by Ca^2+^-dependent kinases at moderate Ca^2+^ signal intensity. Also, nonphosphorylatability of Ser^343/347^ rendered activation of RBOHD by BIK1 Ca^2+^ sensitive (Fig. 4D). While RBOHD^S343/347A^ activation by BIK1 was already substantially reduced at HCa^2+^, this activation level collapsed down to ~20% of that of RBOHD at MCa^2+^ and was almost abolished at RCa^2+^. These findings reveal that Ser^343/347^, when phosphorylated, allows for maximal activation of RBOHD at resting Ca^2+^ concentration (RCa^2+^, 87 nM) solely through phosphorylation of other p-sites, while nonphosphorylated Ser^343/347^ necessitates higher Ca^2+^ concentrations (HCa^2+^, 630 nM) for efficient Ca^2+^ binding to RBOHD EF-hands as alternative means allowing for full NOX activation. In conclusion, the phosphorylation status of Ser^343/347^ likely regulates the Ca^2+^ binding affinity/efficiency of the adjacent EF-hands. Phosphorylation of Ser^343/347^ by either BIK1 or CBL1/CIPK26/CPK5 would therefore be a key step on the substrate level for enabling Ca^2+^-sensitized ROS generation. We also discovered that Ser^343/347^ as well as all other p-sites that are shared between CBL1/CIPK26 and CPK5 displayed substantially enhanced phosphorylation intensity when simultaneously exposed to both kinases as compared to the phosphorylation intensity conferred by individual kinases (Fig. 4A).

**Fig. 5.**
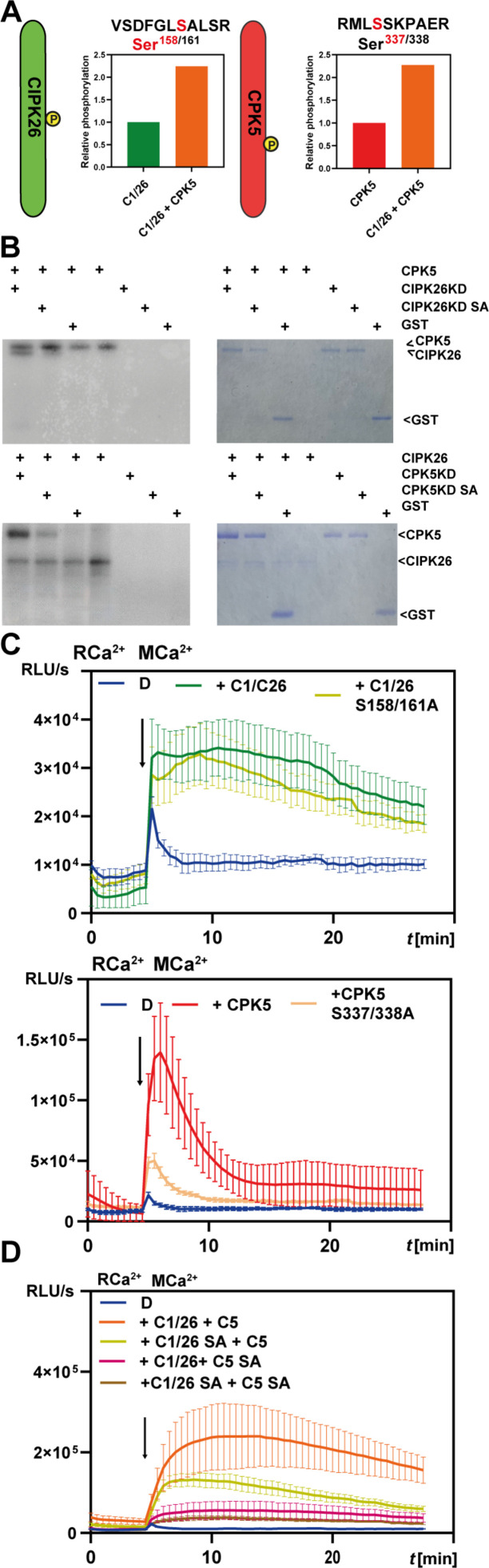
Mutual hyperactivation of CIPK26 and CPK5 through reciprocal phosphorylation. (**A**) Ser^158^ in CIPK26 and Ser^337^ in CPK5 represent differentially phosphorylated p-sites. The bar graphs indicate the mean phosphorylation intensity of four replicates in HEK293T cells at MCa^2+^ expressing either the combination of both kinases or individual kinases. (**B**) CPK5 phosphorylates kinase-inactive CIPK26 in vitro and vice versa. Depicted are autoradiograms and CBB-stained gels. Mutation of the serine residues S158 and S161 to alanine on kinase-inactive CIPK26 reduces the CPK5-mediated phosphorylation of the resulting protein. Similarly, mutation of the two serine residues S337 and 338 to alanine in kinase-inactive CPK5 reduces the CIPK26-mediated phosphorylation of the resulting protein. CIPK26KD: kinase-dead CIPK26^K42N^; CPK5KD: kinase-dead CPK5^D221A^; protein amounts loaded: CPK5 (active): 50 ng, CIPK26 (active): 200 ng, CPK5KD, CPK5KD SA, CIPK26KD, and CIPK26KD SA: 2 μg. (**C**) Impact of p-site mutation on the individual ability of CIPK26 and CPK5 to activate RBOHD. (**D**) Impact of p-site mutation on the combined ability of CIPK26 and CPK5 to activate RBOHD. C1/26, CBL1/CIPK26 complexes; 26 SA, CIPK26^S158/161A^; C5 SA, CPK5^S337/338A^. ROS production was quantified as relative light units. Error bars indicate SD. Each data point represents the mean of six wells analyzed in parallel.

We therefore considered that CBL1/CIPK26 and CPK5 could mutually enhance their capability for substrate phosphorylation by, e.g., mutual trans-phosphorylation, reciprocally enhanced auto-phosphorylation, or both. Inspection of our phospho-proteomic dataset identified Ser^158/161^ in CIPK26 as being principally phosphorylated and displaying a higher degree of phosphorylation upon coexpression with CPK5 (Fig. 5A). Also, in CPK5, we found that intensity of Ser^337/338^ phosphorylation was enhanced by CBL1/CIPK26 coexpression (Fig. 5A). In vitro phosphorylation assays combining inactive CIPK26 (CIPK26KD, harboring a K42N substitution) with active CPK5 established that CPK5 efficiently phosphorylated CIPK26 (Fig. 5B). Vice versa, phosphorylation of inactive CPK5 (CPK5KD, harboring a D221A substitution) by active CIPK26 was also readily detectable. Notably, Ser to Ala conversion of either Ser^158/161^ in CIPK26KD or Ser^337/338^ in CPK5KD diminished the degree of their phosphorylation. This latter observation confirms these amino acids as phosphorylation targets. To molecularly detail the contribution of Ser^158/161^ (in CIPK26) and Ser^337/338^ (in CPK5) to conferring Ca^2+^ sensitization of RBOHD activation, we characterized their potential to activate this NOX at MCa^2+^. Mutation of Ser^158/161^ in CIPK26 (CIPK26^S158/161A^) did not substantially affect its ability to activate RBOHD, while mutation of Ser^337/338^ (CPK5^S337/338A^) reduced the individual capability of CPK5 to activate RBOHD (Fig. 5C). However, reciprocal combinations of nonphosphorylatable with phosphorylatable kinases (CPK5^S337/338A^ + CBL1/CIPK26 and CPK5 + CBL1/CIPK26^S158/161A^) dramatically reduced the synergistic activation of RBOHD when compared to combination of mutually phosphorylatable kinases (CPK5 + CBL1/CIPK26) (Fig. 5D). Here, the effect of CPK5^S337/338^ appeared to be more pronounced than that of CIPK26^S158/161A^, coinciding with the high Ca^2+^ responsiveness (and a low half maximal kinase activity of 100 nM Ca^2+^) of CPK5 (*15*). These data identify mutual phosphorylation of both kinases as being required for Ca^2+^-sensitized RBOHD activation (Fig. 5D). Additionally, combination of both mutant versions (CPK5^S337/338A^ + CBL1/CIPK26^S158/161A^) even further diminished the synergistic RBOHD activation capability normally conferred by combined function of both kinases. Collectively, these results support the importance of mutual phosphorylation of CPK5 and CIPK26 for their ability to confer synergistic activation of RBOHD.

## DISCUSSION

### Emerging principles of Ca^2+^-sensitized RBOHD activation in systemic PTI

Systemic signaling in multicellular organisms involves organ-scale (trans-cellular) as well as organismic (trans-organ) propagation mechanisms. In plants, organismic signals transduce via vasculature wires and mechanistically involve electrical, chemical, and mechanical components, while in animals nerve cords and vasculature provide convergent solutions to serve the dissemination of electrical or hormonal signals (*29*). Trans-cellular signaling within an organ involves diffusion and/or propagation of second messengers and can serve dispersion of incoming signals within an organ but can also precede subsequent trans-organ signal dissemination (*24*, *27*). Mutually interdependent Ca^2+^ and ROS waves crucially function in both organismic and organ-scale signaling (*7*, *21*, *23*). Here, we identify and characterize the CBL1/CIPK26/CPK5 module as functioning in both processes by simultaneously conferring Ca^2+^-sensing and Ca^2+^-dependent phosphorylation for activating the NOX RBOHD, a key enzyme for mounting plant immunity and stress tolerance.

A first important insight emerging from our studies is that all components of the CBL1/CIPK26/CPK5 signaling module crucially contribute to organismic implementation of systemic immunity, because representative PTI responses are impaired in distal leaves after local elicitor application in individual *cipk26* and *cpk5* mutants. Abolishment of PTI in *cipk26/cpk5* establishes the requirement of this module for interorgan proliferation of systemic immunity. The combined outcome of our marker gene induction analysis and Ca^2+^ signaling assays specifies this bi-kinase module as conferring both local (trans-cellular) and systemic (trans-organ) immune responses. We further focused on detailing the molecular mechanisms that define its function in organ-scale manifestation of trans-cellular Ca^2+^/ROS signal propagation. These investigations revealed that the PRR-activated but Ca^2+^-independent kinase BIK1 and the Ca^2+^-activated kinases CBL1/CIPK26 and CPK5 confer phosphorylation of distinct (“Ca^2+^-independent” and “Ca^2+^-dependent”) but also of a shared set of target sites. Of these, the shared Ser^343/347^ p-site apparently brings about Ca^2+^ sensitization of RBOHD. Our experimental data do not allow to distinguish if this results from modulation of the Ca^2+^ binding efficiency of the EF-hands in RBOHD or if the Ser^343/347^ phosphorylation status brings about a conformational change mimicking Ca^2+^ binding to these EF-hands. In line with a central role of Ser^343/347^ as switch for sensitizing RBOHD activation is that Ser^343/347^ are the most strongly phosphorylated residues in the whole Arabidopsis proteome after flg22 treatment (*30*). Another most relevant discovery reported here is the mutual phosphorylation of CPK5 and CIPK26, which causes synergistically enhanced RBOHD substrate activation by both kinases already in response to delicate Ca^2+^ signals (MCa^2+^; 150 nM). This Ca^2+^ sensitization mechanism occurs at concentrations close to the resting level of the cytoplasm (RCa^2+^; 87 nM) and coincides with the high Ca^2+^ sensitivity of CPK5 (and its half maximal kinase activity at 100 nM). The critical Ser^337/338^ site in CPK5 resides in a loop adjacent to the autoinhibitory pseudosubstrate domain of this kinase, while in CIPK26 the critical Ser^158^ residue locates in the DFG +2 position of the activation loop. Remarkably, previous studies on mammalian and yeast kinases have established that the identity and phosphorylation of the respective residues in these kinases modulate their substrate phosphorylation and preference, suggesting a potential mechanism for the mutual activation of CPK5 and CIPK26 (*31*, *32*). This discovery solves the long-standing enigma of how trans-cellular second messenger waves can propagate through organs without interfering with intracellular signaling processes in individual cells along this path. The combined consequence of this two-layer Ca^2+^ sensitization and activity potentiation mechanism reported here allows for organ-scale dispersion of a low-amplitude Ca^2+^ wave (that we detected here in flg22-treated leaves) by “sidling” through individual cells without disturbing ongoing essential intracellular Ca^2+^ signaling processes. Notably, the activity regulating p-sites in CIPK26 and CPK5 is not unique to these kinases but appears to be conserved in several, but not all, members of both kinase families (fig. S5). The apparent conservation of this activation mechanism is suggestive for the occurrence of similar mechanisms in other systemic stress response processes in plants.

**Fig. 6.**
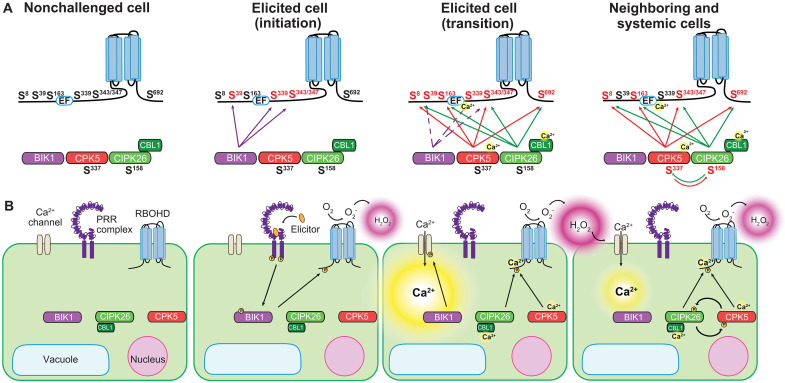
Molecular and cellular models of RBOHD regulation in systemic signal initiation and propagation. (**A**) Succession of phosphorylation events and the resulting phospho-code in RBOHD during different steps of PTI. (**B**) Sequence of events during the initiation and subsequent propagation of immune signaling at the cellular level.

Collectively, our findings allow to deduce a model for switching from initial local to subsequent systemic signaling in innate immunity and for sustaining this long-distance signal after initiation (Fig. 6). In the primary elicitor-exposed cell(s), elicitor binding activates the PRR complex and confers direct activation of specific RLCKs, including BIK1 (*12*, *13*). During defense signaling initiation, BIK1 in turn phosphorylates RBOHD at multiple sites including Ser^343/347^, resulting in NOX activation and local extracellular ROS production. These directly elicitor-stimulated cells also form primary Ca^2+^ signals that may or may not contribute to RBOHD activity via direct EF-hand binding and activation of Ca^2+^-dependent kinases. Paracrine signaling by apoplastic ROS activates Ca^2+^ channels in neighboring cells, allowing for subtle increases in cellular Ca^2+^ concentration, that suffice to activate the CBL1/CIPK26/CPK5 module in the absence of PRR activation. Both kinases synergistically phosphorylate and thereby activate RBOHD for maximal ROS production already triggered through minute elevation of cytoplasmic Ca^2+^ concentration. This allows signal propagation to the next distal cell eventually forming an iterative paracrine cell-to-cell signaling circuit manifesting as propagating Ca^2+^/ROS signal. In this way, the CBL1/CIPK26/CPK5/RBOHD module concomitantly confers organ-scale signaling initially within the primary challenged leaf, but also subsequently throughout the whole plant resulting in systemic immunity.

## MATERIALS AND METHODS

### Plant materials and growth conditions

*A. thaliana* ecotype Columbia-0 (Col-0) was used as WT, and T-DNA insertion lines *cipk26*-2 (GK-703D04), *cipk26*-3 (SALK_005859C), and *cpk5* (SAIL_657_C06) were obtained from the European Arabidopsis Stock Centre (http://arabidopsis.info). *cipk26-2* (GK-703D04) and *cipk26-3* as well as *cbl1/9* were described earlier (*33*–*35*). *rbohd* (SALK JP65_4B03L) was obtained from J. Schroeder (*36*). *cipk26/cpk5* double mutants were generated by crossing of *cipk26-2* and *cpk5*. For Ca^2+^ analysis, plant lines were transformed by the floral-dip method with *Agrobacterium tumefaciens* GV3101 (pMP90) harboring the plasmid pGGZ003-UBI10-R-GECO1-GSL-mTurquoise (*37*).

*A. thaliana* Col-0 WT and derived transgenic overexpressing and mutant plants were grown on soil with 8-hour light/16-hour dark cycle, 23°C, and 60% relative humidity. *Nicotiana benthamiana* plants were grown on soil in a greenhouse with 16-hour light/8-hour dark cycle.

### Bacterial growth assays

Bacterial pathogen *P. syringae* pv. *tomato* DC3000 was grown in King’s B medium at 28°C overnight. For measuring bacterial growth, Arabidopsis leaves of 6-week-old plants were infiltrated with *Pst* DC3000 at 10^4^ colony-forming units (CFU)/ml in 10 mM MgCl_2_ using a needleless syringe. Three days after inoculation, bacterial growth was monitored by serial dilution plating of ground leaf discs.

### ROS measurements in Arabidopsis

ROS production was monitored using a luminol-based assay. Flagellin-dependent oxidative burst in *A. thaliana* was conducted with 6-week-old plants grown under short day conditions. Leaf discs (0.3 cm) were floated overnight on 100 μl of H_2_O in a 96-well plate, and luminescence was recorded using a Berthold Tristar LB941 plate reader.

### Gene expression by qRT-PCR analysis

To analyze transcript levels, RNA was extracted from leaf tissue using the TRIzol method. RNA (2 μg) was treated with ribonuclease (RNase)–free deoxyribonuclease (DNase) (Fermentas) and reverse-transcribed with SuperScript III SuperMix (Invitrogen) according to the manufacturer’s protocols. Real-time quantitative polymerase chain reaction (qRT-PCR) analysis was performed in a final volume of 10 μl according to the instructions of Power SYBR Green PCR Master Mix (Applied Biosystems) using the CFX96 system (Bio-Rad). Amplification specificity was evaluated by post-amplification dissociation curves. *ACTIN2* (At3g18780) was used as the internal control for quantification of gene expression.

### Calcium imaging

#### Growth conditions and sample preparation

For the analysis of Ca^2+^ signals evoked by flg22 in leaves, Arabidopsis plants were germinated on MS solid medium and transferred to soil ~1 week after germination and grown under long-day conditions. True leaves of ~1-cm length were cut off and incubated for 24 hours under constant light in incubation buffer (10 mM MES, 5 mM KCl, 10 mM CaCl_2_, pH adjusted to 5.8 with tris). Subsequently, leaves were mounted upside down on a microscope slide. At the petiole, a twofold barrier consisting of 1% low melting point agarose and a second layer of plasticine was created to separate the leaf blade from the petiole (see Fig. 4B). The purpose of this barrier is to prevent direct contact of the elicitor to the leaf blade. Finally, the sample was covered with a coverslide. After this mounting procedure, samples were again incubated for at least 6 hours under continuous light.

#### Epi-fluorescence image acquisition for measurement of calcium dynamics

For R-GECO1–mTurquoise–based in vivo Ca^2+^ imaging (*37*), an inverted ZEISS Axio observer microscope was used (Carl Zeiss Microimaging GmbH, Goettingen, Germany), which was equipped with a xenon short arc reflector lamp (Hamamatsu), a Zeiss EC Plan-NEOFLUAR 5×/0.16 dry objective, an ET436-20x T455lp ET480-40m filter set for mTurquoise, an ET560-40x T585lpxr ET630-75m filter set for R-GECO1, and a Retiga R6 camera, and operated by Visiview software (Visitron Systems GmbH, Puchheim, Germany).

An exposure time of 400 ms was used for both R-GECO1 and mTurquoise image acquisition with binning 2. Four areas of the leaves were consecutively analyzed every 10 s to cover most of the leaf area. After measurements for 20 cycles of 10 s each, 10 μl of a 4 μM flg22 solution was pipetted to the petiole. Ca^2+^ measurements were then proceeded for 30 min. For each measurement, after application of a Gaussian blur to each image, ratio images of the four leaf areas were combined to ratio stacks, and finally, the four ratio stacks were combined to a single stack using ImageJ. To determine the speed of the Ca^2+^ waves, ROIs with approximate size of an epidermal cell were defined in the proximity to the leaf’s middle vein in each of the four leave areas (covering the length of the leave). The arrival of the Ca^2+^ wave manifested as a maximum in the R-GECO1–mTurquoise ratio. Through determination of the distance between individual ROIs and the time difference between the ratio maxima, the speed of the waves was calculated.

For the kymograms presented in fig. S4, 180-pixel–long (316-μm) line type ROIs were defined in the first and third quarter of the leaves in proximity of the midvein. Kymograms were generated using the Multi Kymograph function of ImageJ. The resulting kymograms were exported as text images, and the ratio of each pixel at each time point was normalized to the pixel’s mean intensity before application of the flg22 stimulus. The resulting normalized kymograms were reimported into ImageJ, and the false color code was unified for all kymograms to allow comparison of ratio changes between the genotypes.

### BiFC analysis

For transient expression in 5- to 6-week-old *N. benthamiana* epidermal cells, *A. tumefaciens* GV3101 (pMP90) carrying BiFC constructs were co-infiltrated with the p19 strain into leaves as described previously (*38*). The yellow fluorescent protein (YFP) C-terminal fragment SPYCE(M) was fused to the N terminus of RBOHD, and the N-terminal YFP fragment SPYNE(R)173 was fused to the N termini of all 26 Arabidopsis CIPKs (*39*). Microscopic analyses of lower epidermal cells were conducted at 3 days after infiltration. An inverted fluorescence microscope, Leica DMI6000B, equipped with a Leica N Plan L 20×/0.4 CORR PH1 objective (Leica, Wetzlar, Germany) and a Hamamatsu Orca camera (model C4742-80-12AG, Hamamatsu Photonics, Shizuoka, Japan) and a YFP filter set, which was operated with Openlab 5.0.2 software (Improvision, Coventry, UK), was used for BiFC quantification at lower magnification. For subcellular localization studies at higher magnification, an inverted confocal laser scanning microscope, Leica DMI6000, equipped with a Leica TCS SP5 II confocal laser scanning device (Leica Microsystems) and a 63×/1.2 water immersion objective (HCX PL APO lambda blue 63.0 × 1.20 Water UV) was used.

### Protein expression for in vitro assays

For protein purification, the coding sequences (CDS) of the substrate proteins RBOHD N terminus (amino acids 1 to 372), the inactive CPK5^D221A^ variant, and the inactive CIPK26^K42N^ were fused with an N-terminal 2xStrepII-GST tag, and the CDS of active CPK5 was fused with an N-terminal 2xStrepII tag in the pET-24b vector (Merck, Darmstadt, Germany). The CDS of active CIPK26 was fused with an N-terminal 2xStrepII tag in the pIVEX-1.3WG vector (Biotechrabbit, Berlin, Germany). CIPK26 was expressed using the cell-free wheat germ RTS 500 system according to the manufacturer’s protocol (Biotechrabbit, Berlin, Germany). Active CPK5 and substrate proteins were induced and expressed in *Escherichia coli* BL21 CodonPlus(DE3)-RIL cells (Stratagene) overnight at 18°C after induction with 1 mM isopropyl-β-D-thiogalactopyranoside (IPTG). Cell pellets were harvested, solubilized using a French press (Avestin EmulsiFlex-C3; ATA Scientific, Taren Point, Australia), and subsequently purified using Strep-Tactin-Macroprep (IBA Lifesciences, Germany). For the preparation of the 2xStrepII-GST-RBOHD-Nt protein, after mechanical cell disruption using the French press, proteins were solubilized from inclusion bodies using 8 M urea (*17*). StrepII-tagged proteins were purified using Strep-Tactin MacroPrep (IBA Lifesciences, Göttingen, Germany) following the manufacturer’s instructions.

Purified proteins (50 ng of CPK5 and 200 ng of CIPK26, and 2000 ng of RBOHDs, 2000 ng of GST, 2000 ng of CIPK26^K42N^, 2000 ng of CPK5^D221A^) were mixed in reaction buffer [0.5 mM CaCl_2_, 5 mM MnSO_4_, 2 mM dithiothreitol, 10 μM adenosine triphosphate (ATP), and 4 μCi of [γ-^32^P]ATP (3000 Ci mmol^−1^)]. Reactions were incubated at 30°C for 30 min, stopped by addition of 6 μl of 5× SDS-loading buffer [125 mM tris-HCl (pH 6.8), 5% (v/v) glycerin, 1% (w/v) SDS, 2.5% (v/v) β-mercaptoethanol, 0.025% (w/v) bromphenol blue], and analyzed by SDS-PAGE (polyacrylamide gel electrophoresis). Protein bands were fixed by Coomassie staining, and γ-^32^P–labeled proteins were visualized by autoradiography.

### ROS measurements in HEK293T cells

Vectors and methods for HEK293T cell transfection used in this study have been previously described (*17*, *40*). The coding sequence of RBOHD was amplified by PCR and integrated into the pEF1-2xStrepII-N vector (*17*) and the pGGHEK vector (*40*). HEK293T cells were seeded into 96-well plates and incubated in Dulbecco’s modified Eagle’s medium (DMEM)/Ham’s F-12 (Fisher Scientific, Pittsburgh, USA) supplemented with 10% fetal bovine serum (FBS) (Fisher Scientific, Pittsburgh, USA) until reaching about 40% confluency. Transfection with plasmids carrying the coding sequences of the indicated proteins was performed using GeneJuice transfection reagent (Merck, Darmstadt, Germany). Forty-eight hours after transfection, ROS measurements were conducted as previously described (*17*). In brief, cells were subjected to a buffer containing horseradish peroxidase and L-012. Ionomycin was added to induce Ca^2+^ influx into the cells. ROS production was detected through luminescence measurements using either a Mithras2 LB943 (Berthold, Bad Wildbad, Germany) or a Tecan SPARK (Tecan, Männedorf, Switzerland) plate reader.

### Ca^2+^ measurements in HEK293T cells using Fura-2

HEK293T cells were seeded into 96-well plates and incubated in DMEM/Ham’s F-12 (Fisher Scientific, Pittsburgh, USA) supplemented with 10% FBS (Fisher Scientific, Pittsburgh, USA) until reaching about 80% confluency. Before the measurement, the medium was aspirated from the cells and replaced with the Fura-2–loading solution [5 μM Fura-2-AM and 0.15% Pluronic F127 (Invitrogen, Waltham, USA) dissolved in DMEM/Ham’s F-12 with 10% FBS]. After 1 hour of incubation at 37°C at 5% CO_2_, the loading solution and cells were washed with HBSS-Ca^2+^, -Mg^2+^ (Fisher Scientific, Pittsburgh, USA). Hanks’ balanced salt solution (HBSS) with indicated Ca^2+^ concentrations was added to the wells. Fura-2 fluorescence was measured with a Tecan Safire-2 plate reader (Tecan, Männerdorf, Switzerland). For the determination of absolute Ca^2+^ concentrations, Fura-2 fluorescence was calibrated with defined Ca^2+^ buffers generated with the Ca^2+^ Calibration Kit #1 (Invitrogen, Waltham, USA).

### Generation of HEK293T protein samples for liquid chromatography–tandem mass spectrometry analysis

HEK293T cells were cultivated in individual T-75 flasks (Sarstedt, Nümbrecht, Germany). At 30 to 50% confluency, cells were transfected with plasmids encoding the respective heterologous proteins using the GeneJuice transfection reagent (Merck, Darmstadt, Germany). After 48 hours of incubation at 37°C at 5% CO_2_, the medium was aspirated, cells were washed with HBSS-Ca^2+^, -Mg^2+^ (Fisher Scientific, Pittsburgh, USA). After 5 min of incubation in HBSS supplemented with 0.125 mM CaCl_2_, 1 μM ionomycin was added to induce Ca^2+^ influx. After 5 min, the medium was aspirated, ice-cold phosphate-buffered saline (PBS) buffer was added, and cells were collected using a cell scraper. After one wash step in ice-cold PBS, cells were pelleted by centrifugation, the supernatant was aspirated, and cell pellets were flash-frozen in liquid nitrogen and stored at −80°C until further protein purification.

### Liquid chromatography–tandem mass spectrometry–based quantitative proteome analyses

Proteins were extracted from cell pellets, and further sample processing and liquid chromatography–tandem mass spectrometry (LC-MS/MS) data acquisition were performed as described previously (*41*). Briefly, proteins were extracted and digested using a modified filter-assisted sample preparation protocol. After reduction and alkylation, proteins were digested using trypsin. For total proteome analysis, 10 μg of each sample was put aside and analyzed without further processing. For phosphopeptide enrichment, 500 μg of peptides was enriched on titanium dioxide (TiO_2_) (*42*). LC-MS/MS analysis was performed by using an EASY-nLC 1200 (Thermo Fisher Scientific, Waltham, USA) coupled to a Q Exactive HF mass spectrometer (Thermo Fisher Scientific, Waltham, USA). Separation of peptides was performed on 20-cm frit-less silica emitters (New Objective, 0.75 μm inner diameter), packed in-house with reversed-phase ReproSil-Pur C_18_ AQ 1.9 μm resin (Dr. Maisch, Ammerbuch-Entringen, Germany). The column was constantly kept at 50°C. Peptides were eluted in 115 min applying a segmented linear gradient of 0% to 98% solvent B (solvent A: 0% acetonitrile (ACN), 0.1% formic acid (FA); solvent B: 80% ACN, 0.1% FA) at a flow rate of 300 nl/min. Mass spectra were acquired in data-dependent acquisition mode according to a TOP15 method. MS spectra were collected by the Orbitrap analyzer with a mass range of 300 to 1759 mass/charge ratio (*m*/*z*) at a resolution of 60,000 full width at half maximum (FWHM), maximum injection time (IT) of 55 ms, and a target value of 3 × 10^6^ ions. Precursors were selected with an isolation window of 1.3 *m*/*z*, and higher-energy collisional dissociation (HCD) fragmentation was performed at a normalized collision energy of 25. MS/MS spectra were acquired with a target value of 10^5^ ions at a resolution of 15,000 FWHM, maximum injection time of 55 ms, and a fixed first mass of *m*/*z* 100. Peptides with a charge of +1, >6, or with unassigned charge state were excluded from fragmentation for MS^2^, and dynamic exclusion for 30 s prevented repeated selection of precursors.

Processing of raw data was performed using the MaxQuant software version 1.6.17.0 (*43*). MS/MS spectra were assigned to the UniProt Homo sapiens reference proteome supplemented with the sequences of the kinases used for transfection. During the search, sequences of 248 common contaminant proteins as well as decoy sequences were automatically added. Trypsin specificity was required, and a maximum of two missed cleavages was allowed. Carbamidomethylation of cysteine residues was set as fixed, and oxidation of methionine, deamidation, and protein N-terminal acetylation was set as variable modifications. A false discovery rate of 1% for peptide spectrum matches and proteins was applied. Match between runs and requantify options were enabled.
