# Supplementary material for: A bi-kinase module sensitizes and potentiates plant immune signaling
Source: Sci Adv. 2025 Jan 24;11(4):eadt9804. doi: 10.1126/sciadv.adt9804 (PMC11759040; doi:10.1126/sciadv.adt9804)
Supplement: Supplementary file 1 — Figs. S1 to S5 Tables S1 and S2 Legend for movie S1 [file sciadv.adt9804_sm.pdf]

Science Advances
AAAS

Supplementary Materials for

A bi-kinase module sensitizes and potentiates plant immune signaling

Philipp Köster *et al.*

Corresponding author: Jörg Kudla, jkudla@uni-muenster.de

Sci. Adv. **11**, eadt9804 (2025)
DOI: 10.1126/sciadv.adt9804

The PDF file includes:

Figs. S1 to S5
Tables S1 and S2
Legend for movie S1

Other Supplementary Material for this manuscript includes the following:

Movie S1

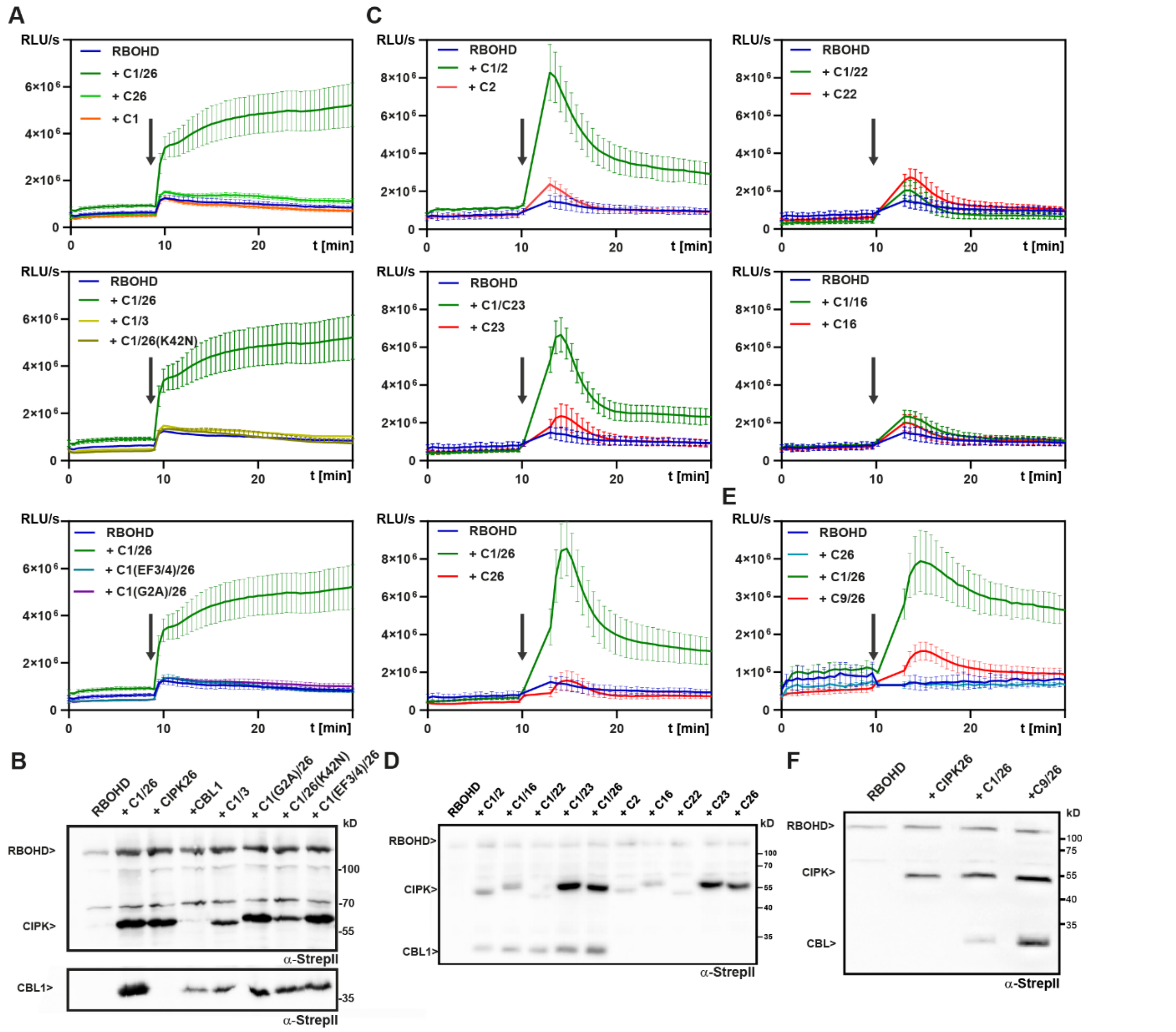

Fig. S1 Kinase activity of CIPK26, CBL1 membrane targeting and Ca^{2+}-binding are required for RBOHD activation. (A) Displayed are individual curves of the samples shown in Fig.1E. Ca^{2+} influx into cells was initiated after 10 minutes (indicated by an arrow). ROS production was quantified as relative light units (RLU). Error bars indicate SD. Each data point represents the mean of 3 wells analyzed in parallel. (B) Western blot indicating expression of the respective proteins from the HEK293T cells assayed in Fig.1E. Total protein extract was analyzed by western blot, StrepII-tagged proteins were visualized using α-StrepII antibody. **C1/26** = CBL1/CIPK26 complex; **C1** = CBL1; **C26(K42N)** = CIPK26K42N; **C1/3** = CBL1/CIPK3 complex. **C1(G2A)** = CBL1G2A**; C1(EF3/4)** = CBL1 EF3/4 (C) In complex with CBL1, CIPK23 and CIPK2, but not CIPK22 or CIPK16 bring about similar RBOHD activation as CBL1/CIPK26 complexes in HEK293T cells. Ca^{2+} influx into cells was initiated after 10 minutes (indicated by an arrow). ROS production was quantified as RLU. Error bars indicate SD. Each data point represents the mean of 5 wells analyzed in parallel. (D) Western blot indicating expression of the respective proteins from the HEK293T cells assayed in Fig.S1C. Total protein extract was analyzed by western blot, StrepII-tagged proteins were visualized using α-StrepII antibody. **C1/26** = CBL1/CIPK26 complex; **C1/2** = CBL1/CIPK2 complex; **C1/16** = CBL1/CIPK16 complex; **C1/22** = CBL1/CIPK2 complex; **C1/23** = CBL1/CIPK23 complex; **C2** = CIPK2; **C16** = CIPK16; **C22** = CIPK22; **C23** = CIPK23; **C26** = CIPK26. (E) CBL1/CIPK26 complexes bring about stronger RBOHD activation than CBL9/CIPK26 complexes. Ca^{2+} influx into cells was initiated after 10 minutes (indicated by an arrow). ROS production was quantified as relative light units (RLU). Error bars indicate SD. Each data point represents the mean of 5 wells analyzed in parallel. (F) Western blot indicating expression of the respective proteins from the HEK293T cells assayed in Fig.S1E. Total protein extract was analyzed by western blot, StrepII-tagged proteins were visualized using α-StrepII antibody. C1/26 = CBL1/CIPK26 complex, **C9/26** = CBL9/CIPK26 complex

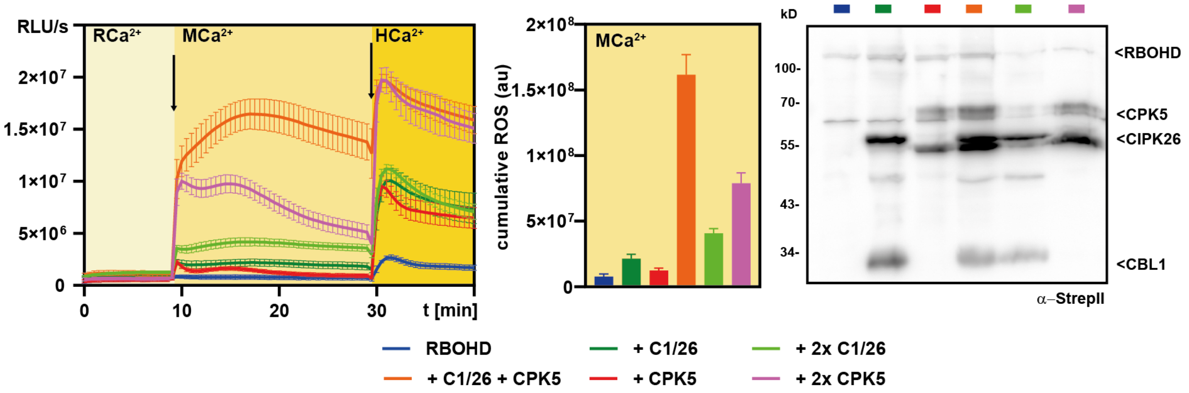

Fig.S2 Synergistic activation of RBOHD by CBL1/CIPK26 and CPK5 does not result from increased expression of individual kinases. Doubling the amount of transfected plasmid DNA of either CBL1/CIPK26 or CPK5 results in additive but not synergistic activation of RBOHD. The measurement curves for RBOHD, RBOHD + C1/26, RBOHD + CPK5, RBOHD + C1/26 + CPK5 are identical to Fig.2D since they result from the same experiment. ROS production was quantified as relative light units (RLU). Error bars indicate SD. Each data point represents the mean of 6 wells analyzed in parallel. Western blot indicating expression of the respective proteins from the HEK293T cells assayed. Total protein extract was analyzed by western blot, StrepII-tagged proteins were visualized using α-StrepII antibody. **C1/26** = CBL1/CIPK26 complexes.

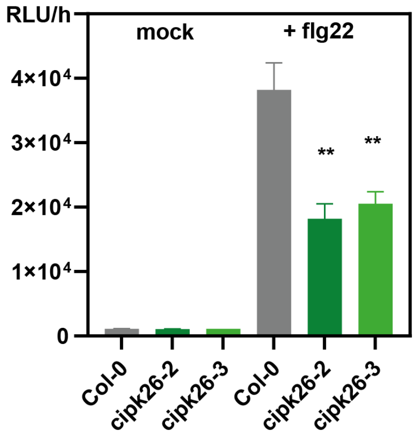

Fig.S3 Two independent mutant alleles of *CIPK26* are impaired in flg22 induced ROS generation. ROS generation was determined over 60 min via a luminol-based assay with and without treatment with 200 nM flg22 in 6-week-old plants of Col-0, *cipk26-2*, and *cipk26-3*. RLU, relative light units; error bars, SEM (n = 16); one-way ANOVA, Dunnett posttest, asterisks denote statistically differences compared to Col-0, **P < 0.01).

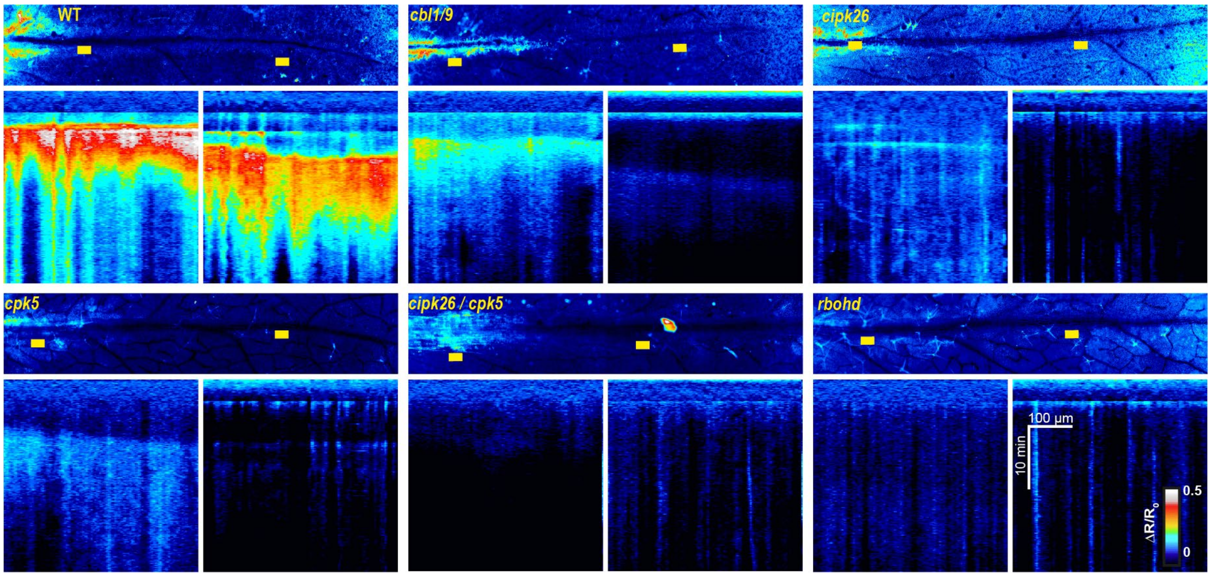

Fig. S4. Propagation of flg22 induced Ca^{2+} signals in mutants of the CBL1/CIPK26/CPK5 axis. Overview pictures and kymograms of representative RGECO-mTurquoise Ca^{2+} measurements which were used to measure the speed of the organ-scale Ca^{2+} waves displayed in Fig.3G. Genotypes and the location of two line type ROIs are indicated in the wide scale overview pictures, which were taken at the beginning of the individual measurements. For each of those 180 pixels long (316 µm) line type ROIs, a kymogram was generated, that displays the $\Delta R/R_0$ ratio change for each of the pixels at every timepoint of the measurements and is displayed as the square image below the line type ROIs.

A

CIPK26	DFGLSALSRQ
CIPK17	DFGLSALSQH
CIPK3	DFGLSALSQQ
CIPK12	DFGLSAVSDQ
CIPK19	DFGLSAVSDQ
CIPK13	DFGLSVVSEQ
CIPK15	DFGLSALSDS
CIPK9	DFGLSAFSRQ
CIPK6	DFGLSAFTEH
CIPK11	DFGLSALTDQ
CIPK14	DFGLSALTDQ
CIPK23	DFGLSALPQQ
CIPK8	DFGLSALPEQ
CIPK24	DFGLSALPQE
CIPK21	DFGLSAVPKS
CIPK1	DFGLSALPQH
CIPK2	DFGLSALADC
CIPK10	DFGLSALADC
CIPK2	DFGLSAMKEQ
CIPK4	DFGLSALPEH
CIPK7	DFGLSALPEH
CIPK16	DFGLSALMMP
CIPK5	DFGLSALPEQ
CIPK25	DFGLSALPEQ
CIPK18	DFGLSAVAEQ
CIPK20	DFGLSALRES

B

CPK5	AKDLIRRMLSSKPAERL
CPK33	AKDLVRRMLTQDPKRRI
CPK9	AKDLVRRMLTADPKRRI
CPK22	AKHLIGKMLTKKPKERI
CPK21	AKDLVRKMLTKDPKRRI
CPK23	AKDLVEKMLTEDPKRRI
CPK15	AKDLVRKLLTKDPKQRI
CPK17	AKDLVKKMLNSDPKQRL
CPK34	AKDLVRKMLNSDPKQRL
CPK12	AKDLIKKMLESNPKKRL
CPK6	AKDLIRKMLCSSPSERL
CPK26	AKNLIRGMLCSRPSERL
CPK20	AKDLVRRMLIRDPKKRM

Figure S5: Alignments of the activating p-sites detected in CIPK26 and CPK5. p-sites in the respective regions within CIPK26 and CPK5 are highlighted in red. CIPK26 Ser158 is strictly conserved in Arabidopsis CIPKs (A), in Arabidopsis CPKs Ser and Thr residues at the respective positions of CPK5 Ser337 and Ser338 are conserved in 12 of 34 isoforms.

TableS1: Phospho-peptides found in CBL1, CIPK26, CPK5, BIK1 and RBOHD proteins after heterologous expression in HEK293T cells and enrichment. For each sample, the intensities detected in each of the four independent replicates are provided.

TableS2. Overview of the Oligos used in this study.

Primers	Sequence (5'——3')
**Primers for plasmid construction, for HEK293T expression**	
CBL1_AvrII_r	tttcctaggtgtggcaatctcatcg
CBL1_BamKoz_f	tttggatccgccgccaccatgggctgcttcc
CBL1_E172Q_f	ggaaaaattgataaattacagtggagtgatttcgtaaac
CBL1_E172Q_r	gtttacgaaatcactccactgtaatttatcaatttttcc
CBL1G2A_BamKoz_f	tttggatccgccgccaccatggcatgcttccactc
CBL9_AvrII_r	tttcctaggcgtcgcaatctcgtcc
CBL9_BamKoz_f	tttggatccgccgccaccatgggttgtttcc
CIPK2_Spe_f	aaaaactagtgagaacaaaccaagtgtattaact
CIPK2_NotI_r	tttgcggccgcctatgatggttcttgctctcc
CIPK3_AvrII_f	tttcctaggaatcggagacagc
CIPK3_NotI_r	ttttgcggccgctcactttgctgtttc
CIPK16_SpeI_f	ttttactagtgaagaatcaaaccgtagtagtac
CIPK16_NotI_r	tttgcggccgctcatgaaacattatttattttgttatcatt
CIPK22_AvrII_f	aaacctagggccgaagactctaattcttcc
CIPK22_NotI_r	tttgcggccgcttacggtttgtcaggaacttt
CIPK23_Spe_f	tttactagtgcttctcgaacaacgccttc
CIPK23_NotI_r	ttttgcggccgcttatgtcgactgttttgcaattgt
CIPK26_AvrII_f	tttcctaggaatcggccaaaggttc
CIPK26_NotI_r	ttttgcggccgcttatttgcttagaccagagc
CIPK26_K42N_f	cgtggctctcaacatacttgataag
CIPK26_K42N_r	cttatcaagtatgttgagagccacg
CPK5_Spe_f	aaaactagtatgggcaattcttgccgtgg
CPK5_Xho_r	aaactcgagcgcgtctctcatgctaatgttta
CPK5_S337/338A_f	atccgcagaatgttagccgccaagcctgcagaacgtttg
CPK5_S337/338A_r	caaacgttctgcaggcttggcggctaacattctgcggat
BIK1_Bam_f	aaaaggatccatgggttcttgcttcagttctcg
BIK1_KpnISpeI_r	aaaaggtaccactagtcacaaggtgcctgccaaaag
RBOHD_AvrII_f	tttcctaggaaaatgagacgagg
RBOHD_NotI_r	ttttgcggccgcctagaagttctctttg
RBOHD_S8A_Spe_f	ttttactagtatgaaaatgagacgaggcaatgcaagtaac
RBOHD_S39A_f	aacaggtctcagccaagaaaaacgcaagattcgccg
RBOHD_S39A_r	aacaggtctcttggccgcacgtttaggccggc
RBOHD_S162/163A_f	gcggtttgaccgcacggccgccgcggccatccacgc
RBOHD_S162/163A_r	ggccgcggcggccgtgcggtcaaaccgc
RBOHD_S343/347A_f	cttgctcagatgttagctcagaagcttagaccggc
RBOHD_S343/347A_r	gccggtctaagcttctgagctaacatctgagcaag
RBOHD_S339A_f	aacaggtctcagccaggatacttagtcagatgttaagtcagaagc
RBOHD_S339A_r	aacaggtctcatggcgtctcccatccgcaccga
RBOHD_S692A_f	aacaggtctcattcgccgaggtttgcaaacctcctac
RBOHD_S692A_r	aacaggtctctcgaaaacggtcctgagcttacgtgtc
pGGA-I_f	cgtatgttgtgtggaattgtg
pGGA-I_r	gctgcaaggcgattaagttg

Primers for plasmid construction, in vitro kinase assay	
RBOHD_Spe_f	tttactagtaaaatgagacgaggcaattc
RBOHD_NTerm_r	tttgaattcttagtcaagtatgaaatatttgattttctc
CIPK26_Spe_f	tttactagtaatcggccaaaggttcagc
CIPK26_Xho_r	tttctcgagttatttgcttagaccagagctctc
CIPK26_S153A_f	gattttggattgagtgcgttg
CIPK26_S153A_r	aacgcactcaatccaaaatccgcgactttcagatttccttg
CIPK26_S158/161A_f	gtctctgattttggattggctgcgttggcccgacaagtcaggggtg
CIPK26_S158/161A_r	cacccctgacttgtcgggccaacgcagccaatccaaaatcagagac
CPK5_SpeI_f	aaaactagtatgggcaattcttgccgtgg
CPK5_SalI_r	aaagtcgacctacgcgtctctcatgctaatg
CPK5_D221A_f	tggtgtgatgcatagagccttgaagcctgagaattt
CPK5_D221A_r	aaattctcaggcttcaaggctctatgcatcacacca
CPK5_S337/338A_f	atccgcagaatgttagccgccaagcctgcagaacgtttg
CPK5_S337/338A_r	caaacgttctgcaggcttggcggctaacattctgcggat
**Primers for RT-PCR**	
cipk26_genotyp.for	gttcttaatgatcaaggttatgac
cpk5_genotyp.for	tcgttccaaattgaccttgac
cpk5_genotyp.rev	gaggaaacagcggagagagac
rbohd_genotyp.for	atgaaaatgagacgaggcaattc
rbohd_genotyp.rev	ggatactgatcataggcgtggctcca
cbl1_genotyp.for	aatctatggaattaggtacaattaggttcggtc
cbl1_genotyp.rev	gtaaacgtaacaaatctttactttttttacttg
cbl9_Xu_for	agcccagcaaagtctgaagtta
cbl9_genotyp.rev	tctagggaagcattaggatgg
LB-SALK	gtgatggttcacgtagtgg
RB-SALK	gagactctaattggataccgag
NHL10_RT_for	ttcctgtccgtaacccaaac
NHL10_RT_rev	ccctcgtagtaggcatgagc
Actin2_RT_for	gtaagagacatcaaggagaagctctc
Actin2_RT_rev	ggagatccacatctgctggaatg

Other Supplementary Material for this manuscript includes the following:

Movie S1. Video of R-GECO fluorescence in an Arabidopsis Col-0 leaf blade after flg22 application to the petiole base. Corresponds to Fig.3 D+E.
